# The Effects of *Lumbricus rubellus* Extract on *Staphylococcus aureus* Colonization and IL-31 Levels in Children with Atopic Dermatitis

**DOI:** 10.3390/medicina59112007

**Published:** 2023-11-15

**Authors:** Meutia Sara, Faridha Ilyas, Kartini Hasballah, Nurjannah Nurjannah, Mudatsir Mudatsir

**Affiliations:** 1 Doctoral Program of Medical Science, Faculty of Medicine, Universitas Syiah Kuala, Banda Aceh 23111, Indonesia; meutiasara@mhs.usk.ac.id; 2 Department of Dermato-Venereology, Faculty of Medicine, Universitas Hasanuddin, Makassar 90245, Indonesia; faridhailyas@unhas.ac.id; 3 Department of Pharmacology, Faculty of Medicine, Universitas Syiah Kuala, Banda Aceh 23111, Indonesia; kartini.hasballah@usk.ac.id; 4 Department of Public Health, Faculty of Medicine, Universitas Syiah Kuala, Banda Aceh 23111, Indonesia; nurjannah_dr@usk.ac.id; 5 Department of Microbiology, Faculty of Medicine, Universitas Syiah Kuala, Banda Aceh 23111, Indonesia

**Keywords:** antiallergic agent, antimicrobial activity, *Lumbricus rubellus*, atopic dermatitis, IL-31

## Abstract

*Background and Objectives*: The ineffective combination of corticosteroids and antibiotics in treating some atopic dermatitis (AD) cases has been concerning. The skin barrier defects in AD ease the colonization of *Staphylococcus aureus* (*S. aureus*), which results in a rise in interleukin-31 (IL-31). *Lumbricus rubellus* (*L. rubellus*) has shown antimicrobial and antiallergic effects but has not been studied yet to decrease the growth of *S. aureus* and IL-31 levels in AD patients. This study aimed to analyze the effect of *L. rubellus* extract in reducing *S. aureus* colonization, the IL-31 level, and the severity of AD. *Materials and Methods*: A randomized controlled trial (RCT) (international registration number TCTR20231025004) was conducted on 40 AD patients attending Dermatology and Venereology Polyclinic, Mother and Child Hospital (RSIA), Aceh, Indonesia, from October 2021 to March 2022. AD patients aged 8–16 who had a Scoring Atopic Dermatitis (SCORAD) index > 25, with total IgE serum level > 100 IU/mL, and had healthy weight were randomly assigned into two groups: one received fluocinolone acetonide 0.025% and placebo (control group) and one received fluocinolone acetonide 0.025% combined with *L. rubellus* extract (Vermint^®^) (intervention group). The *S. aureus* colony was identified using a catalase test, coagulase test, and MSA media. The serum IL-31 levels were measured using ELISA assay, while the SCORAD index was used to assess the severity of and improvement in AD. Mean scores for measured variables were compared between the two groups using an unpaired t-test and Mann–Whitney U test. *Results*: A significant decline in *S. aureus* colonization (*p* = 0.001) and IL-31 (*p* = 0.013) in patients receiving *L. rubellus* extract was found in this study. Moreover, fourteen AD patients in the intervention group showed an improvement in the SCORAD index of more than 35% (*p* = 0.057). *Conclusions*: *L. rubellus* extract significantly decreases *S. aureus* colonization and the IL-31 level in AD patients, suggesting its potential as an adjuvant therapy for children with AD.

## 1. Introduction

Atopic dermatitis (AD) is the most common skin disease affecting adults and children (1–20%) worldwide, primarily reported in Nigeria, England and New Zealand [1]. In Southeast Asia, 1.1% of Indonesian children aged 13–14 and 17.9% of Singaporean children aged 12 have had AD [2]. The Indonesian Pediatric Dermatology Study Group reported that AD ranked first (23.67%) among the top 10 pediatric skin diseases and 10 major hospitals across Indonesia. AD was documented in up to 36% of all dermatitis cases in Indonesia [3].

The imbalance of T helper (Th) 1 and Th2 in the “hygiene hypothesis” has been associated with AD pathophysiology [4]. Th1 is associated with microbial infections and autoimmune diseases, while Th2 is associated with parasitic infections and allergic diseases. Frequent exposure to infectious agents stimulates Th1, which in turn balances Th2. Excessive Th2 has recently been reported to cause a rise in allergy cases in industrialized countries [5,6].

The mechanism underlying AD consists of a complex interaction among skin barrier defects, immune dysregulation, the environment, and infectious agents. The immune and skin barrier deficits in AD predispose a person to bacterial infection. The discovery of microorganisms on the skin surface of AD patients is the main factor that worsens AD. Superficial *S. aureus* infection is the most common infection in AD. Studies showed that 80–100% of AD patients have *S. aureus* colonization on the skin or nose, whereas in healthy individuals, the prevalence is only 5–30% [7,8,9]. *S. aureus* induces T-cell-independent B-cells, upregulate proinflammatory cytokines, such as TSLP, IL-4, IL-12, and IL-22, and mast cell degranulation which produces Th2 and exacerbates skin inflammation [10,11,12].

Antimicrobial agents are often prescribed to control the acute phase of AD. Consequently, bacterial resistance in AD patients increases, complicating the job of the dermatologists to provide the appropriate prescriptions [13,14,15]. The inflammatory process of AD is caused by allergens leading to the skin barrier breakdown and the subsequent exposure of the extracellular matrix to *S. aureus* [16].

Several mechanisms have explained how staphylococcal infection exacerbates inflammation in AD. *S. aureus* also releases superantigen toxins that trigger inflammation by activating superantigen-specific and allergen-specific T cells, which releases specific interleukins, particularly IL-31, a cytokine associated with pruritus [17,18]. Interleukin-31 is an important cytokine in the pathophysiology of AD, playing a role in lesion formation, itching, and neuronal growth. Interleukin-31 acts as a neuro-immune link between Th2 cells and sensory nerves mediating itch [19,20]. Serum IL-31 levels were significantly increased in AD compared to healthy controls and correlated with AD severity. Therapeutic approaches in the IL-31 pathway have been shown to be effective in AD [19,21].

*L. rubellus* or earthworm has been used as a traditional medicine for over 2300 years. *L. rubellus* extract has been studied to balance the Th1 and Th2 cells [22]. *L. rubellus* has also been known for its antibacterial activity against *S. aureus* and contains antihistamines, which reduce pruritus [22,23,24]. In Indonesia, *L. rubellus* extract is widely used and freely available on the market [22,23]. However, studies assessing the effect of *L. rubellus* on the colonization of *S. aureus* and IL-31 serum levels are limited. Considering the currently available AD therapy approaches only relieve AD symptoms, *L. rubellus* might shed light on an alternative and adjuvant AD treatment. This study aims to examine the effect of *L. rubellus* extraxt on *S. aureus* colonization formation and IL-31 levels in children with AD. In addition, this study also aims to explore the effectivity of *L. rubellus* in reducing the severity of AD, measured with SCORAD.

## 2. Materials and Methods

### 2.1. Study Design

This was a single-blind, parallel randomized controlled trial (RCT) assessing the effectivity of *L. rubellus* extract based on the *S. aureus* colonization and IL-31 levels in pediatric AD patients attending the Dermatology and Venereology Polyclinic, Mother and Child Hospital (RSIA), Aceh, from October 2021 until March 2022. The patients who met the inclusion criteria were divided into two groups: those who received fluocinolone acetonide 0.025% and placebo (control group) and those who received Fluocinolone acetonide 0.025% and *L. rubellus* extract (Vermint^®^) (treatment group).

Severity of AD was measured using the SCORAD index, ranged from 0–100. Meanwhile, pruritus intensity was measured by subjective symptom of SCORAD with visual analog scale average for the last 3 days. The serum IL-3 level was measured using an ELISA IgE kit, and was measured in IU/mL. The formation of *S. aureus* colony was measured using the Pour Plate method using Manittol Salt Agar, and was scored from 0 to 3, depending on the number of colonies found. The measurement of study variables (SCORAD index, serum IL-31 level, and *S. aureus* colonization) was carried out at baseline and after 14 days. Measurement of the serum IL-31 level was conducted at the Physiology Laboratory, Faculty of Veterinary Medicine, Universitas Syiah Kuala (USK), while the *S. aureus* colonization was examined at the Microbiology Laboratory, Faculty of Medicine, USK.

This study had been registered into the Thai Clinical Trials Registry, registration number TCTR20231025004. Moreover, this study was also approved by the Ethics Committee of the Faculty of Medicine, USK—Dr. Zainoel Abidin General Hospital, Banda Aceh (Ethical Approval Number: 157/EA/FK-RSUDZA/2021). Prior to data collection, patients’ parents or caregivers were given an explanation about the study procedure, benefit of the study and possible side effects. Signed informed consents from the parents/caregivers who were willing to participate in the study.

### 2.2. Study Population

The inclusion criteria were child (1) AD patients with SCORAD index > 25 and meeting the Hanifin and Rajka criteria, (2) aged 8–16 years old, with a (3) total serum IgE level > 100 IU/mL and a (4) healthy weight (5th–85th percentile) based on the CDC BMI-for-age (2–20 years old) growth chart. Patients with the following criteria were excluded: (1) treated with steroids, systemic antihistamine, or phototherapy in the last month and systemic immunosuppressive therapy in the last three months, (2) obtained a systemic antibiotic in the last one month, (3) used topical antibiotic or antiseptic soap in the last two weeks, (4) had immunosuppressive or systemic diseases, and (5) had positive feces identification for intestinal nematode parasites (e.g., *Ascaris lumbricoides, Enterobius vermicularis*). The drop-out criteria were applied during the study for those who (1) did not turn back for the follow-up session, (2) did not obey the study protocols, (3) experienced serious adverse events, and (4) did not have improvements in the adverse events following the temporary discontinuation of therapy.

### 2.3. Randomization

Patients who met the inclusion criteria were recruited and labelled as odd or even. They were randomized using a computer-generated system (SPSS 29) and divided into two groups: (1) a control group who received 0.025% fluocinolone acetonide and placebo; and (2) an intervention group who received 0.025% fluocinolone acetonide and *L. rubellus* extract (Vermint^®^).

### 2.4. Blinding of Participants

After being assigned to one of the groups, the data collector performed blinding on the patients using a single-blinding method, in which patients did not know in which group they were or which medication they took. Medications given to both groups were designed to have the same color and form. The patients were informed about which medications they took after the treatment.

### 2.5. Study Procedure

Patients’ caregivers (parents/guardians) were asked to fill out a questionnaire about the patients’ identity and history of AD, atopy, asthma, allergic rhinitis, previous/ongoing medications, and other diseases. The clinical examinations included the Hanifin and Rajka criteria diagnosis and AD severity assessment with the SCORAD index [25,26]. Skin lesions were photographed with a 48-megapixel camera (Iphone 14 pro max^®^) at a distance of 20 cm and a white background. Blood samples (5 cc) was obtained from the antecubital vein to assess the serum level of IL-31. Skin lesions were smeared for *S. aureus* culture and identified. Patients were instructed to consume Vermint^®^ 250 mg twice a day for 14 days, followed by applying fluocinolone acetonide 0.025% cream twice a day. A logbook was provided for each patient to note the medication schedule and record any side effects. The patients were evaluated after fourteen days.

### 2.6. Measurement of IL-31 Serum Level

Blood samples were collected in EDTA tubes and centrifuged for 20 min at 2000–3000 rpm speed. The supernatant without sediment was then taken out. The plasma was stored at −80 °C for six months. About 50 μL standard solution was added to the standard well. Then, 40 μL of the sample was added into the sample well followed by 10 μL of antibody anti IL-31. A total of 50 μL streptavidin–HRV was added into the sample well and standard well and mixed. Both solutions were left for 30 min of incubation under a temperature of 37 °C. After 30 min, the plate was washed five times with buffer solution and the wells were left with 0.35 buffer solution for 30 s to 1 min. About 50 μL of A substrate solution and 50 μL of B substrate solution were added into each well and left for another 10 min at 37 °C temperature. Then, 50 μL of stop solution was added into each well, after which the bluish color changed to yellowish. Interleukin-31 serum levels were assessed using a microplate reader at 450 nm within 10 min after the addition of stop solution.

### 2.7. Measurement of S. aureus Colony

The AD lesions were smeared using a sterile swab that had been rinsed with phosphate-buffered saline (PBS) three times and isolated with a pour-plate method no later than four hours. The isolate was cultured in the test tubes filled with Mannitol Salt Agar (MSA) media (Becton Dickinson GmbH, Heidelberg, Germany), which had been sterilized with an autoclave at 121 °C for 15 min. Each test tube was labelled based on each dilution (10^−1^, 10^−2^, 10^−3^, 10^−4^, and 10^−5^). One milliliter of each dilution was added to sterile Petri dishes, followed by 18–20 mL of MSA media, before homogenization. The *S. aureus* colony was incubated at 37 °C for 24–48 h and identified with a catalase and coagulase test.

### 2.8. Statistical Analysis

The data were statistically analyzed using IBM SPSS 29 (IBM, New York, NY, USA). Categoric data (gender) were presented in number and percentage, and numeric parametric data (age, SCORAD index) were presented as the mean ± standard deviation (SD), while non-parametric numeric data (pruritus intensity, the level of IL-31 serum, and number of *S. aureus* colony) were presented as the median and interval. The distributions for normality were assessed using the Shapiro–Wilk test. In bivariate analysis, an unpaired t-test was used to compare the mean between two independent groups. The non-parametric data were analyzed with the Mann–Whitney U test. The significant analysis was statistically accepted at *p* < 0.05.

## 3. Results

Forty AD patients were recruited in this study. There was no significant age difference between both groups (*p* = 0.621), with a predominance of female patients (n = 11). Patients in the control and treatment groups had a history of atopy by 35% and 50%, respectively. The baseline means of AD severity level based on SCORAD in the control group was 50.28 ± 9.3, and the treatment group was 50.51 ± 9.94 (*p* = 0.939). The SCORAD level decreased in the follow-up session, which ranged 36.85 ± 8.15 and 26.05 ± 7.69 in the control and intervention group, respectively (*p* < 0.001). The *S. aureus* colonization also significantly declined following the intervention (*p* = 0.001). The IL-31 level also decreased, but not significantly, after the treatment from 78.97 to 42.05 in the intervention group (Table 1).

This study found a significant difference (*p* = 0.005) in the delta change in the SCORAD index after 14 days, in which the median of the delta change in SCORAD index in the control group was 9, while in the intervention group it was 26, indicating a bigger decrease in the SCORAD index in the treatment group (Table 2).

**Figure 1:**
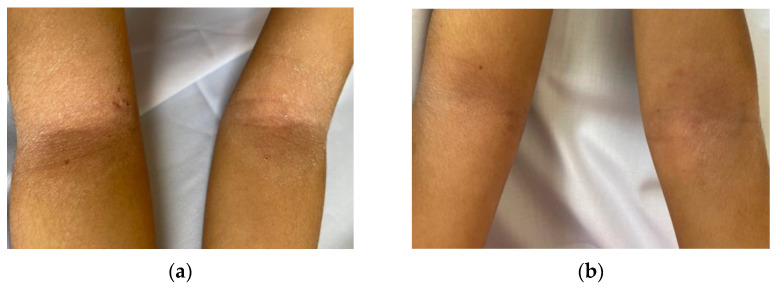
The antecubital fossa of an 8-year-old female patient who received fluocinolone acetonide 0.025% combined with *L. rubellus* extract (intervention group): (**a**) before treatment; (**b**) after 2-weeks of treatment.

Clinical improvement was also observed in intervention group after two weeks of treatment (Figure 1).

A significant difference was also found in the median of the delta change in *S. aureus* colonization (*p* = 0.001), suggesting that the number of the *S. aureus* colony in the intervention group decreased after 14 days (Table 3).

The median of delta change in IL-31 serum levels were 20.41 pg/mL and 34.18 pg/mL in the control and intervention group, respectively. This indicates a significant decrease in IL-31 serum levels (*p* = 0.013) in AD patients treated with *L. rubellus* extract (intervention group) (Table 4).

This study found that 14 patients in the treatment group had an improvement in the SCORAD index of ≥35%, while only 8 patients in the control group had a ≥35% improvement in SCORAD index. However, this difference was not significant statistically (*p* = 0.057). Regardless of the statistical result, this study found that the control event rate (CER) and experiment event rate (EER) were 0.4 and 0.7, respectively. The overall number needed to treat (NNT) was 3.33, rounded up to 4 people, suggesting the number of AD patients receiving the *L*. *rubellus* extract as an additional treatment to increase the SCORAD index (Table 5)

## 4. Discussion

Atopic dermatitis generally occurs in preschool age and is persistent throughout childhood [27]. Approximately 60% of children have AD in the first year of life and 90% by their fifth year. The pathogenesis of AD is complex, comprising genetic, impaired immunologic, and environmental factors affecting the skin barrier. Various skin manifestations, like erythema, oedema, excoriation, crust, and dry skin, occur in AD based on age, chronicity, trigger factors, and skin infections [15,28]. The greatest remission of AD is at the age of 8–11 years, followed by children aged 12–16 years [29]. This was the reason why we included the patient age group 8–16 years in this study. Moreover, assessing subjective complaints of itching and sleep disturbances based on the SCORAD index is easier in children over 7 years of age [30].

This RCT aimed to elucidate the effect of *L. rubellus* extraxt on *S. aureus* colonization formation and IL-31 levels in children with AD, as well as to assess *L. rubellus* effectiveness in reducing the severity of AD, measured with SCORAD.

*S. aureus* is a Gram-positive bacteria found in 20–30% of healthy individuals’ skin and 30–100% in AD patients. A study from Japan reported that impetigo occurred twice as much in AD patients. AD patients’ immune and skin barrier deficits are favorable for *S. aureus* infection. Inflamed skin increases the expression of fibronectin and fibrinogen to which *S. aureus* binds. The increased pH and barrier damage due to skin excoriation cause the excessive growth of *S. aureus*. *S. aureus* toxin activates antigen-presenting cells (APCs) and increases T-cell cutaneous lymphocyte antigen (CLA) expression. The flares in AD cause a shift in the skin microbiome to *S. aureus*. Erosive plaque, yellowish crusts, folliculitis, and abscesses are clinical indicators of bacterial skin infection requiring antibiotics [15,27]. This bacterium produces virulence factors determining pathogenicity and resistance to antimicrobials, including toxins, enzymes, and antigens, allowing bacteria to evade the host’s natural defenses [31]. *S. aureus* is the dominant microbe in AD flares and is associated with the severity of AD [32]. Excoriated inflammatory skin in AD predisposes patients to *S. aureus* colonization, triggering AD recurrence [33]. This corresponds to our finding of the *S. aureus* colony in both groups at baseline.

Topical corticosteroids are the therapeutic of choice for AD. However, *S. aureus* superantigens cause resistant infection to topical corticosteroids by inducing the glucocorticoid *β*-isoform, which has an antagonistic effect on the glucocorticoid receptor *α*-isoform, resulting in T cell resistance to corticosteroids. The combination of antibacterial and corticosteroid treatment was able to control *S. aureus* infection, reduce the attachment of *S. aureus*, and increase the host’s antimicrobial immune response in AD [33]. Unfortunately, some studies have reported the resistance of *S. aureus* to antibiotics such as erythromycin, azithromycin, clarithromycin, and cefuroxime [34,35,36]. *S. aureus* also activates superantigen-specific and allergen-specific T cells, causing the release of specific interleukins, especially IL-31, a cytokine associated with pruritus [31]. The nitrogen components of *L. rubellus* are potentially reported as antihistamines, which might indicate the earthworm to treat pruritus [37]. Pruritus, one of the significant symptoms of AD, has been related to quality of life measured by SCORAD. Staphylococcal toxins induce the secretion of IL-31 through CD4+ T cells in AD patients, hence AD exacerbation [32]. A study revealed that the rise in IL-31 is associated with sleep disturbances and AD severity [38]. A systematic review also coined IL-31 as an important mediator of pruritus in AD. IL-31 will exerts its role in inflammation, pruritus, immune defense and tissue homeostasis by binding with either heterodimer receptors IL-31 receptor A (IL31RA) or oncostatin M receptor beta subunit (OSMRβ) [39]. This study found that *L. rubellus* extract reduced IL-31 levels by 34.81 pg/mL compared to the control group (*p* = 0.013). Furthermore, we also found an improvement in the SCORAD index in the intervention group, reflecting improvement in clinical symptoms of AD, including pruritus. Altogether, these findings indicate the potential benefit of *L. rubellus* extract in AD treatment.

*L. rubellus* is an earthworm producing a proline-rich antimicrobial peptide, or lumbricin-I, to inhibit the growth of bacteria, including *S. aureus* [40,41,42]. The peptide forms pores in the bacterial cell wall and impairs cytoplasm stability and metabolites. The bacterial cell deterioration induces cell lysis [40,43]. The antimicrobial peptide scarcely triggers antibiotic resistance, which has made the peptide a promising future antibiotic compared to conventional ones [44,45]. The position of the hydrophilic and hydrophobic side and the secondary structure help the peptides to be effective over the resistant infectious cases. Nuclease, the active component of *L. rubellus*, treats inflammatory diseases and is involved in the innate immune system [46]. The *L. rubellus* extract is indicated to reduce the size of *S. aureus* colonization through antibacterial and anti-inflammatory pathways. In this study, we identified a significant improvement in patients receiving fluocinolone acetonide 0.025% cream combined with oral *L. rubellus* extract 250 mg twice a day for 14 days, compared to those with fluocinolone acetonide 0.025% cream and placebo (*p* = 0.001).

The SCORAD index is a valid, consistent, and comprehensive score, including the intensity and extent of clinical symptoms as well as the severity of AD symptoms. It has adequate interobserver reliability and qualified interpretability. SCORAD is recommended to assess the severity of AD objectively, especially in clinical trials [26,27]. Moreover, the SCORAD index has been associated with bacterial infection in adults with AD [47]. In this study, an improvement in the SCORAD index of >35% was observed more in the intervention group (n = 14 vs. n = 8), but was not statistically significant (*p* = 0.057). Improvement in the SCORAD index of >35% is considered clinically important [48]. Thus, although statistically not significant, the finding of this study suggested that treatment with *L. rubellus* extract results in a clinically important improvement in AD severity.

This study has several limitations. First, the population size was relatively small, which lessened the variance of AD manifestations, patients’ ages, and outcomes following the treatments. Second, the therapy period was limited to only two weeks, which might obscure the identification of further outcomes in a more extended treatment period. Third, only one class of medium-potency corticosteroid cream was applied in this study, which might bias the potencies of other corticosteroids when combined with *L. rubellus* extract. Overall, the baseline of SCORAD, the Hanifin and Rajka criteria, and children’s growth in our study’s population were similar in the given range. Lastly, we cannot preclude that the initial AD manifestations and demographic variance between the control and intervention group, even though it not statistically different, might have contributed to this study’s result due to the small sample size.

## 5. Conclusions

Topical corticosteroids and antibiotics have shown ineffective treatment in several AD cases. *L. rubellus* extract can be combined with corticosteroids to treat AD without the concern of antibiotic resistance and more severe skin manifestations. Our study showed that the *L. rubellus* extract and fluocinolone acetonide 0.025% cream effectively degraded *S. aureus* colonization and downregulated IL-31 in AD patients. However, this combination did not significantly improve the SCORAD index. A critical trial with a bigger sample size is warranted to further examine the potential effect of *L. rubellus* in AD treatment.

## Figures and Tables

**Table 1:** Demographics and clinical characteristics of the included patients.

Characteristics	Control Group (n = 20)	Intervention Group (n = 20)	*p*-Value
Age, (mean ± SD), y	11.1 ± 2.26	10.75 ± 2.17	0.621
Gender, n (%)			0.327
Male	9 (45)	6 (30)	
Female	11 (55)	14 (70)	
History of atopy, n (%)			0.795
Food allergy	7 (35)	6 (30)	
Allergic rhinitis	3 (15)	4 (20)	
Bronchial asthma	4 (20)	6 (30)	
None	6 (30)	4 (20)	
SCORAD index at baseline, mean ± SD	50.28 ± 9.3	50.51 ± 9.94	0.939
SCORAD index at the end of the study, mean ± SD	36.85 ± 8.15	26.05 ± 7.69	<0.001 *
Pruritus intensity at the baseline (subjective), median (min–max)	6 (4–8)	6 (4–8)	0.901
Pruritus intensity at the end of the study (subjective), median (min–max)	4 (2–6)	2 (0–5)	0.003 *
Number of *S. aureus* colony at the baseline, median (min–max)	2 (0–3)	2 (0–3)	0.806
Number of *S. aureus* colony at the end of the study, median (min–max)	1 (0–3)	0 (0–1)	0.001 *
IL-31 level at baseline, median (min–max)	73.12 (46.82–258.1)	78.97 (45.15–297.28)	0.685
IL-31 level at the end of the study, median (min–max)	49.4 (30.67–231.51)	42.05 (25.95–173.97)	0.185

* statistically significant.

**Table 2:** Delta change in SCORAD index after 14 days.

SCORAD Index	Median	Min–Max	*p*-Value *
Control, n = 20	9	1.4–36.4	0.005
Intervention, n = 20	26	7.2–41.7	

* Mann–Whitney u test.

**Table 3:** Delta change in number of *S. aureus* colony after 14 days.

*S. aureus* Colonization	Median	Min–Max	*p*-Value
Control, n = 20	1	0–2	0.001 *
Intervention, n = 20	2	0–3	

* Mann–Whitney u test.

**Table 4:** Delta change in IL-31 serum levels after 14 days.

IL-31 Serum Levels	Median	Min–Max	*p*-Value
Control, n = 20	20.41	9.95–68.17	0.013 *
Intervention, n = 20	34.18	9.56–124.24	

* Mann–Whitney u test.

**Table 5:** Improvement in SCORAD index after treatment.

Group	SCORAD Index Improvement ≥ 35%	SCORAD Index Improvement < 35%	*p*-Value
Control, n = 20	8 (20%)	12 (30%)	
Intervention, n = 20	14 (35%)	6 (15%)	0.057 *

* Chi-square.

## Data Availability

All data generated during this study are included in this published article.
